# Haemodynamic characteristics of thin-walled regions in intracranial aneurysms: intraoperative imaging and CFD analysis

**DOI:** 10.1007/s00701-025-06660-y

**Published:** 2025-09-06

**Authors:** Haveena Anbananthan, Phani Kumari Paritala, Jessica Benitez Mendieta, Han Yu, Tiago Guerzet Sardenberg Lima, Zoe Dettrick, Ee Shern Liang, Alan Coulthard, Zhi-Yong Li, Craig Winter

**Affiliations:** 1 School of Mechanical, Medical and Process Engineering, Queensland University of Technology (QUT), Brisbane, QLD 4000 Australia; 2 Centre for Biomedical Technologies, Queensland University of Technology (QUT), Brisbane, QLD 4000 Australia; 3 Institute for Molecular Bioscience, The University of Queensland, St Lucia, QLD 4072 Australia; 4 Faculty of Health, Queensland University of Technology (QUT), Kelvin Grove, QLD 4059 Australia; 5 Department of Medical Imaging, Royal Brisbane and Womens Hospital (RBWH), Herston, QLD 4006 Australia; 6 Faculty of Medicine, The University of Queensland, Herston, QLD 4006 Australia; 7 Faculty of Sports Science, Ningbo University, Ningbo, Zhejiang 315211 China; 8 Kenneth G Jamieson Department of Neurosurgery, Royal Brisbane and Womens Hospital (RBWH), Herston, QLD 4006 Australia

**Keywords:** Intracranial aneurysm, Thin-walled region, Computational fluid dynamics, Haemodynamics

## Abstract

**Background:**

Identifying haemodynamic factors associated with thin-walled regions (TWRs) of intracranial aneurysms is critical for improving pre-surgical rupture risk assessment. Intraoperatively, these regions are visually distinguished by a red, translucent appearance and are considered highly rupture prone. However, current imaging modalities lack the resolution to detect such vulnerable areas preoperatively. This study aimed to determine whether thin-walled regions exhibit distinct local haemodynamic profiles compared to adjacent normal-appearing wall regions.

**Methods:**

Sixteen patient-specific models of unruptured middle cerebral artery aneurysms were reconstructed from digital subtraction angiography images. Intraoperative TWRs were identified using a colour segmentation method based on Delta E metrics. Computational fluid dynamics (CFD) simulations were used to compute six haemodynamic parameters: wall shear stress (WSS), time-averaged WSS (TaWSS), oscillatory shear index (OSI), relative residence time (RRT), WSS divergence (WSSD), and pressure. Haemodynamic data were extracted from spatially localised surface patches within confirmed thin and normal regions. Linear mixed-effects models were applied to compare parameters while accounting for patient-level and intra-patient variability, using normalised values to improve model fit.

**Results:**

Thin regions exhibited significantly higher WSS, TaWSS, WSSD, and pressure, and reduced RRT. WSS and TaWSS were approximately 3.3% and 2.8% higher in TWRs, respectively. WSSD was 5.4% higher and RRT was 0.3% lower, suggesting faster, more divergent flow in thin regions. Pressure was modestly but significantly elevated at + 1.3%. No significant difference was observed in OSI between regions.

**Conclusions:**

Thin-walled regions in intracranial aneurysms demonstrate a distinctive haemodynamic profile characterised by stronger, sustained shear forces, greater shear divergence, and reduced residence time, suggesting a dynamic mechanical environment that promotes focal wall thinning. Our findings suggest that persistent shear-driven stress, rather than oscillatory flow, is a key haemodynamic feature of thin-walled regions and may contribute to localised aneurysm wall vulnerability.

**Supplementary information:**

The online version contains supplementary material available at 10.1007/s00701-025-06660-y.

## Introduction

Intracranial aneurysms (IAs) are estimated to affect approximately 2 to 7% of the population and are often discovered incidentally [39]. Although aneurysms can remain asymptomatic, rupture can lead to subarachnoid haemorrhage, which has high rates of morbidity and mortality [25]. Clinical interventions such as microsurgical clipping aim to prevent rupture but carry their procedural risks [16, 35]. One key concern during surgery is the presence of thin-walled regions (TWRs), which appear as red, translucent areas on the aneurysm sac and are considered highly rupture-prone [21].

While size and shape are commonly used as indicators of rupture risk, small aneurysms with localised wall thinning have also been shown to rupture [5, 13, 26, 37]. This suggests that focal wall properties, rather than global aneurysm metrics, may better indicate vulnerability. However, thin regions are currently only identified during surgery. Preoperative imaging lacks the resolution to capture these regions, limiting the ability to assess procedural risk or inform treatment decisions.

To address this challenge, several studies have explored the use of computational fluid dynamics (CFD) to identify flow-related parameters of thin walls [22]. Early work by Kadasi et al. (2013) suggested that thin regions correspond to areas of low wall shear stress (WSS) [13]. In contrast, Sugiyama et al. (2013) found that low WSS was more associated with thick, atherosclerotic regions [28]. Subsequent studies have continued to report conflicting trends, with some identifying high WSS in thin regions [3, 9], while others found the opposite trend [6, 7, 12]. Five studies [14, 15, 24, 30, 32] found no significant correlation between WSS and wall appearance. A recent review by Rajabzadeh-Oghaz et al. (2022) summarised these findings and demonstrated the lack of consensus in this area [21].

A possible explanation for these inconsistencies is the substantial variability in intraoperative image analysis methods [22]. Many studies relied on manual delineation of wall regions, introducing inter-operator subjectivity and limiting reproducibility [38]. In addition, most statistical comparisons treated individual regions as independent observations, despite multiple regions being drawn from the same patient. This overlooks the hierarchical structure of the data and may result in inaccurate estimates or overstated significance.

To overcome these limitations, this study applies a structured and reproducible approach to investigate the flow-wall relationship. We hypothesise that thin-walled regions exhibit distinct local haemodynamic characteristics compared to nearby areas in the aneurysm wall with a normal arterial appearance. CFD simulations were performed on patient-specific aneurysm geometries with clearly visible thin regions identified in intraoperative images. A colour-based method was used to define both thin and normal regions objectively, and flow parameters were extracted from small, spatially confined surface patches centred on manually selected coordinates located well within the boundaries of each region. The data were analysed using linear mixed-effects models (LMMs) to account for the nested structure within patients. This approach aims to provide a more reliable characterisation of the haemodynamic features associated with thin-walled regions.

## Methods

### Ethics and patient consent

This study was conducted in accordance with the Declaration of Helsinki, the National Statement on Ethical Conduct in Human Research (2007, updated 2018), and the Australian Code for the Responsible Conduct of Research (2007). Ethical approval was obtained from the Royal Brisbane and Women’s Hospital Human Research Ethics Committee (RBWH HREC, EC00172) (ref: LNR/2019/QRBW/49363) and the Research Governance and Integrity team at Queensland University of Technology (ref: ID/2490).

As this study involved retrospective analysis of fully de-identified patient imaging data, the requirement for individual informed consent was formally waived by RBWH HREC and the Queensland Department of Public Health (ref: Public Health Agreement #49363).

### Imaging data

Intraoperative images and DSA scans of 16 middle cerebral artery (MCA) aneurysms with clearly defined red regions on the aneurysm sac were included. All patients underwent microsurgical clipping between January 2008 and January 2022 at RBWH. The mean patient age was 61 ± 8.6 years, with 13 female and 3 male patients.

### Thin region identification (Delta E Extraction)

Red regions, defined as translucent areas on the aneurysm wall, were assumed to correspond to thin-walled regions [21]. To objectively identify these regions in intraoperative images, the Delta E metric was applied following the method described by Cho et al. (2018) [6]. This approach quantifies colour differences in the CIELAB colour space, allowing consistent detection of pixels that closely match a manually selected reference colour.

Before segmentation, images were pre-processed using contrast enhancement and manual masking to isolate the aneurysm and parent vessels from the surrounding background tissue. While this improved colour analysis by reducing visual noise, it did introduce some subjectivity. Future implementations could benefit from automated segmentation to improve reproducibility.

**Fig. 1:**
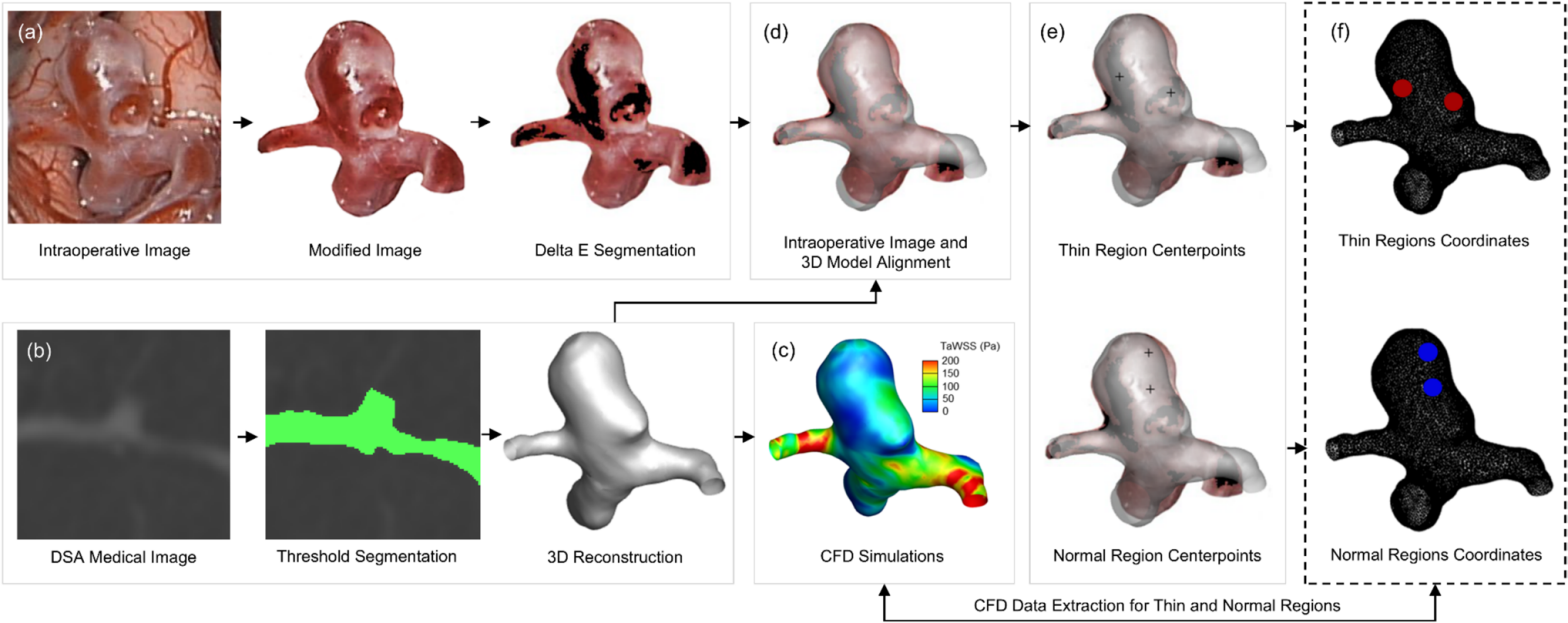
Example of the process to extract haemodynamic data from thin and normal regions of the aneurysm wall in one patient. **a** The processing of the original intraoperative image with Delta E detection of the red (thin) region in black. **b** Segmentation of DSA scans to construct the 3D aneurysm model. **c** CFD (TaWSS) results displayed on the aneurysm model. **d** Overlay of the 3D model with Delta E-marked intraoperative image. **e** Selection of two centerpoints within the thin region and two within the normal region. **f** Points representing thin and normal regions on the aneurysm wall

For each case, a reference red region was then selected at the aneurysm dome. An open-source MATLAB (The MathWorks Inc., Natick, Massachusetts) tool [11] was then used to compute the Delta E between each image pixel and the average CIELAB value of the reference region. A user-defined threshold specified the acceptable colour deviation, enabling segmentation of all pixels within a narrow colour similarity range. This threshold was set to a fixed value of 10 across all cases, based on histogram outputs generated by the tool. As the observed Delta E range in intraoperative images typically spanned up to ~ 80, this threshold corresponded to approximately 12.5% of the maximum colour difference, ensuring only closely matching pixels were included. The threshold was applied relative to each patient’s selected reference colour, allowing consistent identification of similarly red regions. This patient-specific, colour-guided approach enabled the semi-automated segmentation of thin-walled areas without the need to trace every region manually. The final segmentation output was reviewed and confirmed by the operating clinicians to ensure alignment with intraoperative observations. Figure 1(a) illustrates this workflow.

The colour-guided algorithm occasionally captured additional red-hued areas on portions of the parent vessel due to pixel-level matching. However, only regions located on the aneurysm dome and well within the segmentation mask were considered for data extraction. Sampling points were conservatively placed away from the mask boundaries to avoid ambiguous areas and artefacts. Further detail on data sampling is provided in the *Image Co-Registration and Data Selection and Extraction* section.

### Aneurysm geometry reconstruction

Patient DSA scans were segmented using Amira (FEI, Hillsboro, Oregon, USA). Aneurysms and connected arteries were extracted using pixel intensity thresholding, and 3D surface reconstructions were created. This process is shown in Fig. 1(b).

### Computational simulation

The reconstructed geometries were imported into ANSYS SpaceClaim (ANSYS Inc, Canonsburg, Pennsylvania) to define the fluid domain. Inlet and outlet vessels were extended by 15 mm to ensure fully developed flow and prevent recirculation. Tetrahedral volume meshing was performed in ANSYS Meshing (ANSYS Inc, Canonsburg, Pennsylvania) using a body-fitted approach and adaptive refinement. A mesh size of 0.15 mm was selected based on a mesh independence study. A no-slip boundary condition was applied with a 10-layer inflation zone and a growth factor of 1.2 at the inlets and outlets [20].

ANSYS Fluent (ANSYS Inc, Canonsburg, Pennsylvania) was used to perform pulsatile blood flow simulations. Blood was modelled as a homogeneous, incompressible, Newtonian fluid with a density of 1050 kg/m^3^ and a dynamic viscosity of 0.00345 Pa·s [18]. The Navier–Stokes equations were solved for conservation of mass and momentum. Due to the absence of patient-specific velocity profiles, the inlet boundary condition was a time-dependent mass flow rate curve reported by Paritala et al. (2023) [20]. A constant outlet pressure of 80 mmHg was applied.

Each simulation ran for two cardiac cycles, with 3826 time steps per cycle and a time step size of 0.0005 s. Haemodynamic data from the second cycle were extracted to ensure steady-state conditions. Tecplot 360 (Tecplot Inc., Bellevue, WA, USA) was used to extract WSS, time-averaged WSS (TaWSS), oscillatory shear index (OSI), relative residence time (RRT), WSS divergence (WSSD) and pressure. An example TaWSS contour is shown in Fig. 1(c).

The haemodynamic parameters were calculated using the following equations (Eq. 1, Eq. 2, Eq. 3 and Eq. 4), where T is the cardiac cycle duration, s is the location on the artery wall, and t is the time instant.

1 $$TaWSS=\frac{1}{T}{\int }_{0}^{T}\left|WSS\left(s,t\right)\right| dt$$

2 $$OSI=\frac{1}{2}\left(1-\frac{\left|{\int }_{0}^{T}WSS\left(s,t\right)dt\right|}{{\int }_{0}^{T}\left|WSS\left(s,t\right)\right|dt}\right)$$

3 $$RRT=\frac{1}{TaWSS\bullet (1-\left(2\bullet OSI\right)}$$

4 $$WSSD=\frac{{\partial WSS}_{x}}{\partial x}+\frac{{\partial WSS}_{y}}{\partial y}+\frac{{\partial WSS}_{z}}{\partial z}$$

### Image Co-registration and data selection and extraction

Pre-processed intraoperative images were overlaid onto the 3D aneurysm models in Tecplot 360 for spatial alignment, as shown in Fig. 1(d). The CFD model was manually rotated and translated to match the orientation of the image, using arterial landmarks and aneurysm morphology as anatomical reference points [39]. Final alignment was confirmed by the operating clinicians based on intraoperative imaging context and procedural orientation.

To reduce alignment uncertainty from intraoperative deformation or brain shift, sampling regions were conservatively selected well within the boundaries of confirmed thin-walled areas. Red regions identified via Delta E segmentation were used as spatial guides, but only those located on the aneurysm dome and consistent with the intraoperative assessment were eligible for sampling. A similar approach was applied when selecting the comparison/control regions, which were visually identified as normal in appearance and matched the pinkish-purple tone of healthy arterial tissue.

For each patient, two centre points were selected from thin-walled areas and two from normal-coloured areas, resulting in four distinct sampling sites per aneurysm, as shown in Fig. 1(e). Coordinates for each centre point were extracted using Tecplot 360, and an in-house MATLAB script retrieved the ten nearest CFD nodes surrounding each selected coordinate. This ensured a robust local sample around each selected surface point. In total, 40 data points per patient were collected, 20 from thin regions and 20 from normal regions, as shown in Fig. 1(f). At each node, values for WSS, TaWSS, OSI, RRT, WSSD and pressure were extracted for analysis.

### Statistical analysis

Linear mixed-effects models (LMMs) were used to analyse differences in haemodynamic parameters between thin-walled and normal regions while accounting for the nested structure of the dataset. Models were implemented using the MATLAB *fitlme* function from the Statistics and Machine Learning Toolbox.

Several LMM formulations were explored to determine the most appropriate structure for the haemodynamic data, which included repeated measurements from thin and normal regions within each patient. Specifically, two model structures were considered.

$$\mathbf{M}\mathbf{o}\mathbf{d}\mathbf{e}\mathbf{l}1: Y \sim RegionType + (1|Patient) + (1|RegionType)$$

$$\mathbf{M}\mathbf{o}\mathbf{d}\mathbf{e}\mathbf{l}2: Y \sim RegionType + (1|Patient) + (1|Patient|RegionNo)$$

Model 1 assumed independent variability between patients and across regions, whereas Model 2 accounted for clustering of multiple regions ($RegionNo$) within each patient, enabling random variation at both the patient and region-within-patient levels.

Model 2 was selected as the final structure, as it best accounted for the hierarchical nature of the data, where multiple region-level measurements ($RegionNo$) were nested within each patient. This approach allows region-specific variability to differ across patients, improving the accuracy and generalisability of the fixed effect estimates. The general form of the model is shown in Eq. 5 below.

5 $${Y}_{ij}={\beta }_{0}+{\beta }_{1}\bullet {RegionType}_{ij}+\left(1|{PatientID}_{i}\right)+\left(1|PatientID:{RegionNo}_{ij}\right)+{\epsilon }_{ij}$$

where ${Y}_{ij}$​ represents the haemodynamic parameter of interest for patient $i$ at region $j$, ${\beta }_{0}$ is the intercept representing the overall mean, ${\beta }_{1}$ is the fixed effect of the region type. ${RegionType}_{ij}$ is a binary variable (thin or normal), $\left(1|{PatientID}_{i}\right)$ models inter-patient variability and $\left(1|PatientID:{RegionNo}_{ij}\right)$ accounts for random effects at the region level within each patient ${\epsilon }_{ij}$ is the residual error term.

Haemodynamic parameters were analysed using three transformations: raw values, log-transformed values, and values normalised by patient-specific maximum. Model fit was evaluated using a combination of Akaike Information Criterion (AIC) values and residual diagnostics, including Q-Q plots, residuals versus fitted values, histograms, and plots of residuals by observation order. These diagnostics assessed model normality, homoscedasticity, and independence.

Regression coefficients were interpreted according to transformation type. For normalised variables, coefficients represent differences between thin and normal regions in units of standard deviation (SD) from each patient’s maximum. Statistical significance was evaluated at p < 0.05 and 95% confidence intervals were reported for all fixed effects.

## Results

**Fig. 2:**
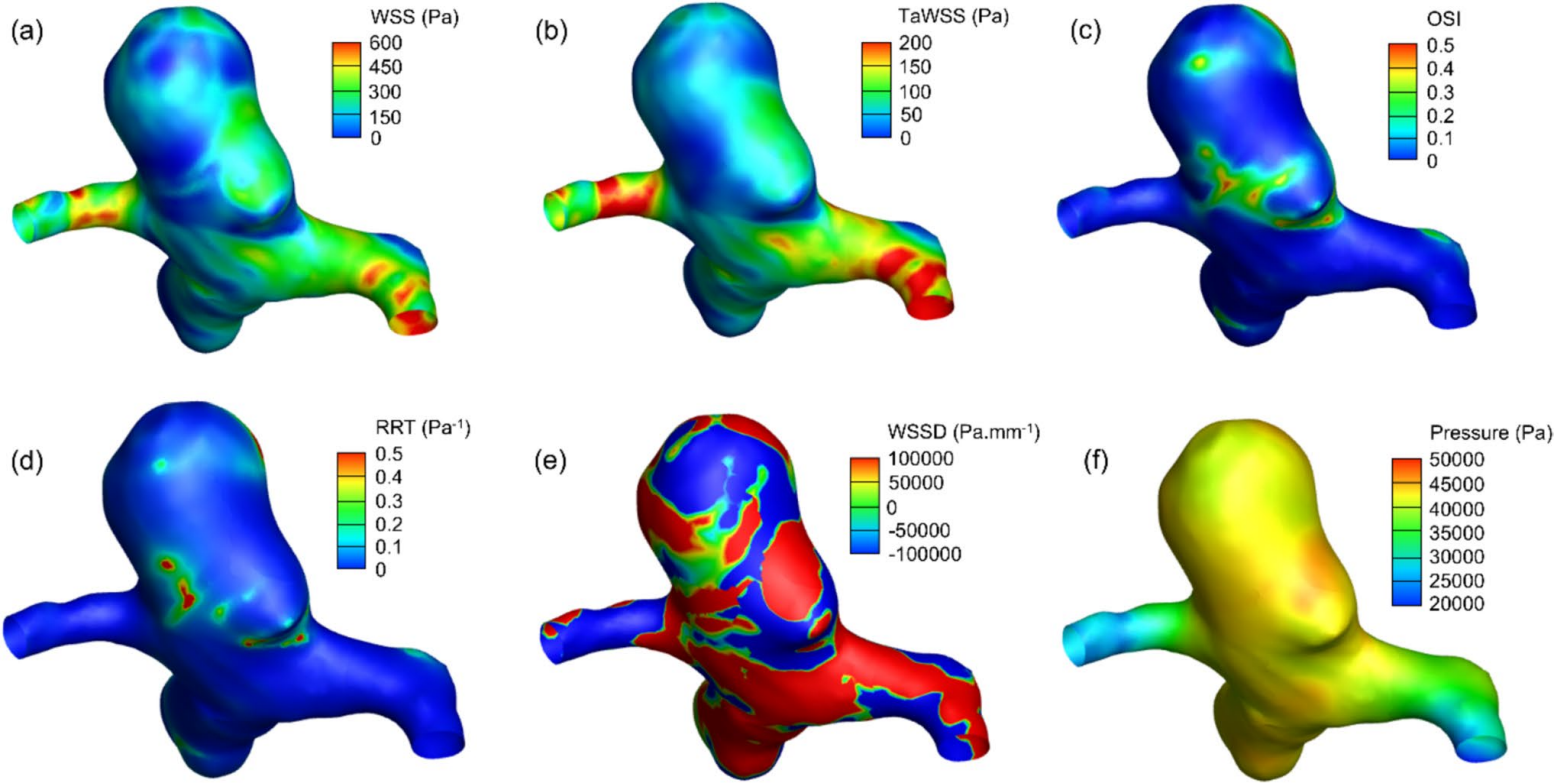
Computational fluid dynamics (CFD) result contours for one patient at systole, showing **a** wall shear stress (WSS), **b** time-averaged WSS (TaWSS), **c** oscillatory shear index (OSI), **d** relative residence time (RRT), **e** WSS divergence (WSSD) and **f** pressure

Computational fluid dynamics (CFD) simulations were used to calculate six haemodynamic parameters across patient-specific aneurysm models. Representative distributions for one patient at systole are shown in Fig. 2, and group-level summary statistics for raw values are reported in Table 1.

**Table 1:** Mean ± standard deviation of raw haemodynamic parameters in thin-walled regions and normal regions across all patients (*n* = 16)

Parameters	Thin Regions	Normal Regions
WSS (Pa)	48.74 ± 42.55	38.34 ± 34.83
TaWSS (Pa)	10.03 ± 9.25	8.06 ± 6.67
OSI	0.05 ± 0.06	0.05 ± 0.08
RRT (Pa^−1^)	0.25 ± 0.25	0.28 ± 0.31
WSSD (Pa.mm^−1^)	66,788.35 ± 268,216.07	− 35,954.87 ± 174,276.64
Pressure (Pa)	15,945.05 ± 7942.13	15,613.53 ± 7346.78

Mean values of WSS and TaWSS were greater in thin regions. OSI and RRT were similar between the two region types. WSSD showed a positive mean in thin regions and a negative mean in normal regions. Pressure values were comparable between the two groups.

As mentioned in the *Methods*, LMMs were then used to quantify differences in haemodynamic parameters between thin-walled and normal regions while accounting for repeated measures within patients. Across the 16 patients, a total of 640 datapoints were analysed, with 320 sampled from thin regions and 320 from normal regions. Final models included fixed effects for region type and random intercepts for patient and region-within-patient.

Model performance was then compared across the three transformation approaches described in the *Methods*: raw values, log-transformed values, and values normalised by patient-specific maximum. Across all parameters tested, models using normalised values consistently demonstrated superior fit. This was evident both visually, through improved residual symmetry and homoscedasticity, and quantitatively, via substantial reductions in AIC values.

For instance, WSS model fit improved from a raw AIC of 3733.7 to − 1645.2 with normalisation. Similarly, TaWSS AIC values improved from 3733.7 to − 1722.6, RRT from − 203.5 to − 4172.6, WSSD from 17 179 to − 1388.5, and pressure from 9879.4 to − 3050.7. These reductions indicate improved overall model fit and more consistent residual behaviour when normalised values were used. Supplementary Information S1 provides a representative example of residual diagnostics across transformation types for the WSS model.

The only exception was OSI, which was analysed in its original form. As OSI is a bounded and already scaled parameter, it could not be normalised, and its raw data residuals showed good distribution without transformation (AIC =  − 2059.3), so no further adjustment was needed.

Based on the final models using normalised haemodynamic parameters, statistically significant differences (p < 0.001) were observed between thin-walled and normal regions for WSS, TaWSS, RRT, WSSD and pressure. OSI did not show a significant difference between region types. All fixed effect coefficients are expressed in units of standard deviation (SD) from each patient’s maximum value, allowing direct interpretation of the relative magnitude of regional differences. Table 2 summarises the estimated effect sizes and 95% confidence intervals for the significant parameters.

**Table 2:** Haemodynamic differences between thin and normal regions of the aneurysm wall, based on LMM results using normalised parameters

Parameter	Normal Region Mean (Intercept)	Thin-Normal Difference (Coefficient)	Thin Region Mean (Intercept + Coefficient)	Effect Size (95% CI)
WSS	0.140	+ 0.033	0.173	0.023 to 0.043
TaWSS	0.141	+ 0.028	0.169	0.019 to 0.037
RRT	0.014	− 0.003	0.011	− 0.004 to − 0.002
WSSD	− 0.017	+ 0.054	0.037	0.042 to 0.066
Pressure	0.908	+ 0.013	0.921	0.01 to 0.016

All values were scaled by the patient-specific maximum, yielding a 0 to 1 range. The table reports the estimated mean in normal regions (intercept), the modelled difference in thin regions (coefficient), the resulting mean in thin regions (sum of intercept and coefficient), and the 95% confidence interval (CI) for the fixed effect. Only parameters with statistically significant differences (*p* < 0.001) are included.

Thin regions exhibited significantly higher normalised WSS compared to normal regions, with a fixed effect coefficient of 0.033 (95% CI: [0.023 to 0.043], p < 0.001). Since all WSS values were normalised by the patient-specific maximum (i.e., scaled from 0 to 1), this coefficient represents a 3.3% increase in thin regions relative to each patient’s maximum WSS. The model intercept of 0.140 corresponds to the average normalised WSS in normal regions, indicating that thin regions reached an average of approximately 0.173 (i.e., 0.140 + 0.033). This reflects a consistent regional effect observed within patients. Random effects analysis further revealed meaningful variability, with estimated variance of 0.023 for patient-level intercepts and 0.059 for patient-region interactions, suggesting individual-specific and region-specific influences on WSS levels.

Thin regions also exhibited significantly higher normalised TaWSS compared to normal regions, with a fixed effect coefficient of 0.028 (95% CI: [0.019 to 0.037], *p* < 0.001). This represents a 2.8% increase in TaWSS relative to each patient’s maximum value. The model intercept of 0.141 reflects the average normalised TaWSS in normal regions, suggesting that thin regions reached a mean value of approximately 0.169 (i.e., 0.141 + 0.028). Patient-level variability contributed a random intercept variance of 0.035, and region-level variability within patients was estimated at 0.053, reflecting modest heterogeneity across subjects and sampled regions.

Normalised RRT was significantly lower in thin regions, with a fixed effect coefficient of − 0.003 (95% CI: [− 0.004 to − 0.002], *p* < 0.001), indicating a small but statistically significant reduction relative to normal regions. The model intercept of 0.0141 represents the average normalised RRT in normal regions, meaning thin regions had a mean value of approximately 0.0111. Random effects analysis showed limited variability, with a patient-level intercept variance of 0.003 and a region-level variance of 0.008, suggesting relatively consistent effects across subjects and sampled regions.

Normalised WSSD was significantly higher in thin regions, with a fixed effect coefficient of 0.054 (95% CI: [0.042 to 0.066], *p* < 0.001), corresponding to a 5.4% increase relative to normal regions. The model intercept of − 0.0175 reflects the average normalised WSSD in normal regions. Random effects analysis showed negligible variance at the patient level (standard deviation ≈ 0), indicating minimal inter-patient variability. However, there was moderate variation across regions within patients, with a standard deviation of 0.075. The residual standard deviation was estimated at 0.075, indicating similar variability at the residual level.

Normalised pressure was significantly higher in thin regions, with a fixed effect coefficient of 0.013 (95% CI: [0.010 to 0.016], *p* < 0.001), equivalent to a 1.3% increase. Random effects variance was 0.052 for patient intercepts and 0.027 for region-level effects within patients, indicating that some variation in pressure values could not be fully explained by differences between patients or regions.

No significant difference was observed for OSI between thin and normal regions, with a fixed effect coefficient of − 0.005 (95% CI: [− 0.012 to 0.002], *p* = 0.17). Although the fixed effect was not statistically significant, the random effects indicated notable variability between patients (variance = 0.053) and across regions within patients (variance = 0.027), suggesting that individual and regional factors still contributed meaningfully to OSI variation.

## Discussion

This study aimed to test whether thin-walled regions of intracranial aneurysms exhibit distinct local haemodynamic characteristics compared to adjacent normal-coloured areas. Thin regions were characterised by significantly higher WSS and TaWSS, lower RRT, and slightly elevated pressure, while OSI did not differ significantly. These findings suggest that thin-walled areas are exposed to a specific haemodynamic environment, dominated by persistent shear stress and faster flow, rather than oscillatory or stagnant flow. This distinctive profile likely contributes to localised wall remodelling and thinning.

Thin-walled regions exhibited a haemodynamic profile characterised by elevated instantaneous and cumulative shear stress. In absolute terms, WSS and TaWSS were higher in thin regions (48.7 ± 42.6 Pa and 10.0 ± 9.3 Pa, respectively) compared to normal regions (38.3 ± 34.8 Pa and 8.1 ± 6.7 Pa). Linear mixed-effects modelling using normalised values confirmed statistically significant differences: WSS was 3.3% higher and TaWSS 2.8% higher in thin regions relative to the patient-specific maximum. These differences were consistent across patients and highlight a pattern of locally intensified shear exposure on thinner segments of the aneurysm wall.

Although the WSS difference appears modest, sustained elevations in shear stress have been implicated in aneurysm wall weakening. Elevated WSS has previously been associated with destructive remodelling processes, including mural cell-mediated changes and increased matrix metalloproteinase activity, collectively degrading the extracellular matrix and weakening the arterial wall [1, 19, 27]. Small but sustained increases in WSS may trigger these pathological processes and progressively compromise aneurysm wall integrity.

The observed relationship between WSS and TaWSS further emphasises the biomechanical stress experienced by thin regions. While WSS represents the instantaneous shear force exerted by blood flow on the endothelial surface, TaWSS reflects average shear stress exposure over the cardiac cycle [17]. The concurrent elevation of both parameters suggests that thin regions are subject to not just transient shear spikes but prolonged mechanical stress.

These findings align closely with prior research by Suzuki et al. (2015), who demonstrated a direct association between high WSS and aneurysm wall thinning [30]. Similarly, Cebral et al. (2018) identified high WSS as a driver of unidirectional collagen fibre alignment [4]. This compensatory mechanism may temporarily reinforce the wall but also generate anisotropic stress distributions, increasing the risk of focal failure [4]. Subsequent work by Cebral et al. (2019) reinforced this association, demonstrating that elevated WSS near flow impingement sites correlated with localised wall thinning [3].

RRT, a marker of disturbed flow indicating areas of low WSS magnitude and high oscillation [23], was significantly lower in thin regions. In absolute terms, mean RRT was 0.25 ± 0.25 Pa^−1^ in thin regions versus 0.28 ± 0.31 Pa^−1^ in normal regions. Linear mixed-effects modelling of normalised data confirmed a statistically significant difference. Thin regions exhibited a 0.003 unit decrease in normalised RRT compared to normal regions (95% CI: [− 0.004 to − 0.002], *p* < 0.001).

This finding reinforces the notion that persistent shear-driven flow, rather than disturbed or stagnant flow, directly contributes to aneurysm wall thinning. RRT is sensitive to areas where WSS is low and fluctuates in direction, which are commonly linked to thicker or atherosclerotic aneurysm walls [28, 29]. In contrast, the lower RRT observed in thin regions suggests a more streamlined, faster flow environment that reduces particle residence time. This may accelerate wall degradation by continuously exposing endothelial cells to sustained haemodynamic stress, impairing their function. The consistency of this result across patients and sampled regions further supports its relevance as a potential marker of remodelling activity in thinning aneurysm walls.

WSSD captures both the magnitude and directional changes of wall shear stress on the aneurysm wall [14]. WSSD was significantly higher in thin-walled regions. In absolute terms, mean WSSD was 66,788 ± 268,216 Pa/mm in thin regions compared to − 35,955 ± 174,277 Pa/mm in normal regions, reflecting marked differences in shear stress directionality. Linear mixed-effects modelling of normalised data confirmed a statistically significant difference, with thin regions exhibiting a 0.054 unit increase in normalised WSSD relative to normal regions (95% CI: [0.042 to 0.066], *p* < 0.001).

This result supports growing evidence that WSSD is a sensitive indicator of wall instability and focal remodelling in intracranial aneurysms. Elevated WSSD has been shown to co-localise with surgically confirmed thin wall regions and has outperformed conventional WSS in predicting zones of mechanical vulnerability [14, 38]. Mechanistically, high WSSD reflects areas of strong flow impingement or divergence, where spatially concentrated tensile shear forces act on the endothelium wall [8]. The minimal patient-level variability observed in WSSD supports the robustness of this marker across different aneurysms. Taken together, the findings highlight WSSD as a promising, flow-derived indicator of structural weakening in intracranial aneurysms.

The combined pattern of elevated WSS, TaWSS, and WSSD, along with reduced RRT, defines a distinctive haemodynamic environment associated with thin-walled regions. These areas appear to be exposed not only to stronger and more sustained shear forces, but also to spatially varying shear fields such as divergence or impingement zones. Such complex flow patterns can create internal stretching and stress gradients on the aneurysm wall, triggering endothelial dysfunction, cellular turnover, and maladaptive remodelling processes including matrix degradation and wall thinning. Among all parameters evaluated, WSSD showed the most robust and consistent association with thin regions, exhibiting both the largest relative difference and minimal inter-patient variability. This highlights WSSD as a particularly promising indicator of local wall instability, capable of capturing the dynamic mechanical cues that precede structural weakening. These findings support the emerging view that fast, impinging, and shear-divergent flow patterns are central to aneurysm wall degeneration and suggest that combining multiple WSS-derived metrics may improve regional risk assessment in future studies [38].

Despite the observed associations between thin-walled regions and shear-related parameters (WSS, TaWSS, WSSD) and flow acceleration markers (lower RRT), no significant difference was found in OSI. OSI, a dimensionless parameter quantifying directional WSS change throughout the cardiac cycle [6]. In absolute terms, OSI was 0.05 ± 0.06 in thin regions and 0.05 ± 0.08 in normal regions. Linear mixed-effects modelling confirmed this result, with a fixed effect coefficient of − 0.005 (95% CI: [–0.012 to 0.002], *p* = 0.17), indicating no statistically significant difference.

This outcome aligns with previous findings by Suzuki et al. (2015), who similarly reported no association between OSI and wall thinning in intracranial aneurysms [30]. The lack of OSI differences may indicate that oscillatory shear stress is less relevant to sustained wall thinning than persistent shear forces. While OSI captures oscillatory shear direction rather than magnitude or spatial distribution, its lack of differentiation here suggests that directional flow reversal may play a limited role in aneurysm wall thinning, at least in the context of MCA bifurcation aneurysms.

Pressure was modestly but significantly elevated in thin-walled regions. In absolute terms, mean pressure was 15 945 ± 7 942 Pa in thin regions compared to 15 614 ± 7 347 Pa in normal regions. Linear mixed-effects modelling of normalised data confirmed this difference, with a fixed effect coefficient of + 0.013 (95% CI: [0.010 to 0.016], *p* < 0.001), indicating a 1.3% increase in thin regions relative to each patient’s maximum.

While the magnitude of this difference is small compared to shear-based metrics, even slight increases in luminal pressure can influence tensile stress on the aneurysm wall, particularly through effects on vascular smooth muscle cells and fibroblasts involved in extracellular matrix remodelling [10]. This aligns with findings by Kadasi et al. (2013), who reported elevated pressures in thin-walled aneurysm areas [13], and subsequent studies by Suzuki et al. (2016) [32] and Tanaka et al. (2019) [34] confirming these associations in larger samples.

Observed variability in haemodynamic parameters across and within patients reinforces the importance of patient-specific modelling approaches. The hierarchical structure captured in the LMM suggests that individual vascular anatomy and flow dynamics may influence local haemodynamic stress and associated wall thinning.

This study's strengths include its structured and reproducible methodology, which addresses several limitations found in earlier investigations. An objective Delta E segmentation framework was used to identify the thin regions on the intraoperative image, and LMMs were used to properly account for the nested data structure, capturing both inter- and intra-patient variability. These steps were taken to mitigate the subjectivity and statistical oversights that have limited previous studies.

Importantly, these methodological refinements also offer a possible explanation for the inconsistent findings reported in the literature regarding the haemodynamic correlates of wall thinning. Conflicting associations have been observed between wall appearance and key parameters such as WSS and OSI, with some identifying high WSS in thin regions [3, 30], others reporting low WSS [6, 7, 13, 33], and several finding no association [14, 15, 31, 32]. These inconsistencies may stem from key methodological differences, including subjective wall classification, lack of standardised sampling, and failure to account for intra-patient correlation.

Beyond methodology, anatomical heterogeneity may also contribute to these varying results. Prior studies have included aneurysms from multiple locations, each with distinct geometry, inflow patterns, and wall structure that may influence haemodynamic forces differently. In contrast, our study focused exclusively on middle cerebral artery bifurcation aneurysms, reducing shape-related variability and enabling more direct regional comparisons. By standardising both the anatomical context and statistical framework, our approach may help reconcile prior inconsistencies and contribute to a clearer understanding of the haemodynamic environment associated with thin-walled regions.

However, several limitations should be acknowledged. The study included a relatively small sample size of 16 unruptured MCA bifurcation aneurysms, which may limit generalisability to aneurysms at other anatomical locations, such as ICA sidewall or anterior communicating artery aneurysms. Larger studies encompassing broader anatomical representation are necessary to draw more definitive conclusions [19].

The identification of thin regions based on intraoperative images introduces potential uncertainties due to indirect wall thickness assessment and variations in lighting or camera angles. Although a standardised Delta E colour metric was used to improve consistency, the segmentation algorithm operates purely on pixel colour similarity and does not account for anatomical context. Hence, red-hued regions may occasionally be detected on the parent artery, particularly if blood or glare introduces similar colour signatures. To mitigate this, all final segmentations were confirmed intraoperatively by the operating clinicians as representing visibly thin, translucent zones, and only regions on the aneurysm dome were selected for haemodynamic analysis. To minimise the inclusion of artefactual or noisy regions, sampling points were conservatively placed well within the central areas of confirmed thin-walled zones, deliberately avoiding boundaries or ambiguous regions. Despite these precautions, future work may benefit from semi-automated or AI-based classification methods that incorporate contextual image features, such as shape, texture, or vascular geometry, to enhance the accuracy and reproducibility of thin-wall identification.

In addition, the image registration process relied on manual alignment between intraoperative images and 3D CFD models using anatomical landmarks and visual orientation, with final confirmation provided by the clinical team. While this approach was guided by procedural context, it remains inherently subjective. Future studies could reduce alignment variability by implementing objective image registration techniques, such as mutual information-based matching or surface registration algorithms, potentially incorporating intraoperative navigation data to support reproducible mapping between imaging and CFD domains.

Furthermore, the CFD analysis assumed blood to be a Newtonian, incompressible, and laminar fluid, potentially oversimplifying complex intra-aneurysmal flow dynamics [36]. Boundary conditions were based on generic literature-derived waveforms rather than patient-specific measurements, introducing another potential source of inaccuracy. Moreover, aneurysm walls were modelled as rigid, ignoring deformation from pulsatile flow. Future research could enhance model accuracy by incorporating patient-specific flow conditions and wall compliance through dynamic mesh techniques [2].

Additionally, haemodynamic parameters were normalised using the maximum value from the entire vascular model, including the parent vessel. While this approach enabled consistent within-patient scaling, it may slightly influence the normalised range observed at the aneurysm dome. Future work could explore dome-specific normalisation to assess sensitivity to this assumption.

Thin-walled regions of intracranial aneurysms exhibited a distinct local haemodynamic profile characterised by elevated and sustained shear stress, faster blood flow, and slightly increased pressure. These findings suggest that persistent haemodynamic stress may contribute to progressive wall thinning and local structural vulnerability. They also emphasise the value of patient-specific flow analysis and highlight the need for continued refinement of computational models to improve rupture risk assessment and support surgical decision-making.

## Supplementary information

Below is the link to the electronic supplementary material.

ESM1 (DOCX 292 KB)

## Data Availability

The data that support the findings of this study are available from the corresponding author upon reasonable request.

## Abbreviations

IA: Intracranial aneurysm
CFD: Computational fluid dynamics
LMM: Linear mixed-effects model
AIC: Akaike Information Criterion
WSS: Wall shear stress
TaWSS: Time-averaged WSS
OSI: Oscillatory shear index
RRT: Relative residence time
WSSD: WSS divergence
