# Supplementary material for: Haemodynamic characteristics of thin-walled regions in intracranial aneurysms: intraoperative imaging and CFD analysis
Source: Acta Neurochir (Wien). 2025 Sep 6;167(1):238. doi: 10.1007/s00701-025-06660-y (PMC12414043; doi:10.1007/s00701-025-06660-y)
Supplement: Supplementary file 1 — (DOCX 292 KB) [file 701_2025_6660_MOESM1_ESM.docx]

**ORIGINAL RESEARCH**

**Haemodynamic Characteristics of Thin-Walled Regions in Intracranial Aneurysms: intraoperative imaging and CFD analysis**

Haveena N. Anbananthan, Phani Kumari Paritala, Jessica Benitez Mendieta, Han Yu, Tiago Guerzet Sardenberg Lima, Zoe Dettrick, Ee Shern Liang, Alan Coulthard, Zhi-Yong Li, Craig D. Winter

From the School of Mechanical, Medical and Process Engineering, Queensland University of Technology (QUT), Brisbane, Queensland, Australia (**HNA, PPK, JBM, TGSL, HY, ZL**), Centre for Biomedical Technologies, Queensland University of Technology (QUT), Brisbane, Queensland, Australia (**HNA, PPK, JBM, TGSL, HY, ZL**), Institute for Molecular Bioscience, The University of Queensland, Brisbane, Queensland, Australia (**JBM**), Research Methods Group, Faculty of Health, Queensland University of Technology (QUT), Brisbane, Queensland, Australia (**ZD**), Department of Medical Imaging, Royal Brisbane and Women's Hospital, Queensland Health, Herston, Queensland, Australia (**ESL, AC**), Faculty of Medicine, The University of Queensland, Herston, Queensland, Australia (**ESL, AC, CW**), Faculty of Sports Science, Ningbo University, Ningbo, Zhejiang, China (**ZL**), Kenneth G Jamieson Department of Neurosurgery, Royal Brisbane and Women's Hospital (RBWH), Brisbane, Queensland, Australia (**CW**).

Please address correspondence to Zhi-Yong Li, PhD (Lond), MA (Cantab), School of Mechanical, Medical and Process Engineering, Queensland University of Technology (QUT), Brisbane, 4000, Queensland, Australia; e-mail: zhiyong.li@qut.edu.au

**ORCID IDs**

Haveena N. Anbananthan: 0000-0003-4193-948X

Jessica Benitez Mendieta: 0000-0001-9778-6158

Han Yu: 0009-0002-9896-9639

Tiago Guerzet Sardenberg Lima: 0000-0001-9342-8804

Zoe Dettrick: 0000-0003-1091-1820

Alan Coulthard: 0000-0003-1110-9207

Zhi-Yong Li: 0000-0002-6814-9165

Craig D. Winter: 0000-0003-4626-7280

## **Abstract**

BACKGROUND. Identifying haemodynamic factors associated with thin-walled regions of intracranial aneurysms is critical for improving pre-surgical rupture risk assessment. Intraoperatively, these regions are visually distinguished by a red, translucent appearance and are considered highly rupture prone. However, current imaging modalities lack the resolution to detect such vulnerable areas preoperatively. This study aimed to determine whether thin-walled regions exhibit distinct local haemodynamic profiles compared to adjacent normal-appearing wall regions.

METHOD. Sixteen patient-specific models of unruptured middle cerebral artery aneurysms were reconstructed from digital subtraction angiography images. Intraoperative TWRs were identified using a colour segmentation method based on Delta E metrics. Computational fluid dynamics (CFD) simulations were used to compute six haemodynamic parameters: wall shear stress (WSS), time-averaged WSS (TaWSS), oscillatory shear index (OSI), relative residence time (RRT), WSS divergence (WSSD), and pressure. Haemodynamic data were extracted from spatially localised surface patches within confirmed thin and normal regions. Linear mixed models were applied to compare parameters while accounting for patient-level and intra-patient variability, using normalised values to improve model fit.

RESULTS. Thin regions exhibited significantly higher WSS, TaWSS, WSSD, and pressure, and reduced RRT. WSS and TaWSS were approximately 3.3% and 2.8% higher in TWRs, respectively. WSSD was 5.4% higher and RRT was -0.3% lower, suggesting faster, more divergent flow in thin regions. Pressure was modestly but significantly elevated at +1.3%. No significant difference was observed in OSI between regions.

CONCLUSIONS. Thin-walled regions in intracranial aneurysms demonstrate a distinctive haemodynamic profile characterised by stronger, sustained shear forces, greater shear divergence, and reduced residence time, suggesting a dynamic mechanical environment that promotes focal wall thinning. These results support the hypothesis that persistent shear-driven stress, rather than oscillatory flow, underlies localised aneurysm wall vulnerability, highlighting the importance of patient-specific CFD analysis in rupture risk assessment.

## **Supplementary Information**

**Supplementary Data S1: Example Residual Diagnostics for Haemodynamics Data Modelling**

This supplementary section presents residual diagnostic plots for the wall shear stress (WSS) model under three transformation types: raw, log-transformed, and normalised. The plots include residuals versus fitted values, residuals versus observation order, a histogram of residuals, and Q-Q plots. These diagnostics were used to assess model assumptions and guide the selection of the optimal data transformation for statistical analysis.

**(A) WSS Model [AIC = 3733.7]**


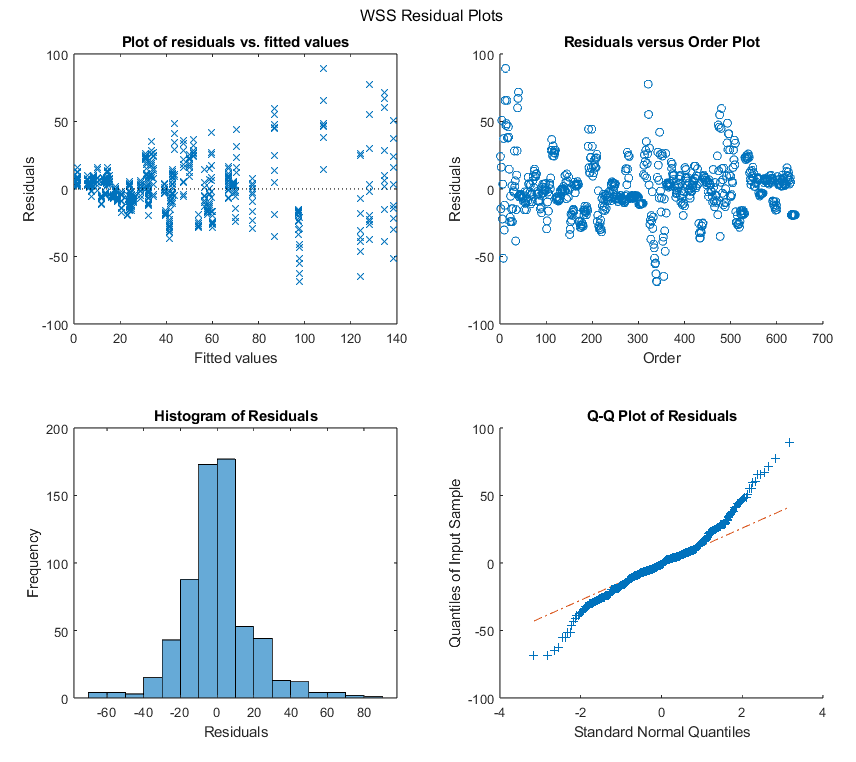


Figure S1.A. Residual plots for the WSS model using raw (untransformed) values. The residuals vs. fitted values plot shows heteroscedasticity, and the Q-Q plot indicates deviation from normality. The histogram suggests moderate skewness. These patterns suggest the model assumptions are not fully satisfied under raw scaling. **AIC = 3733.7**

**(B Log-scale-WSS Model [AIC = 1101.9]**


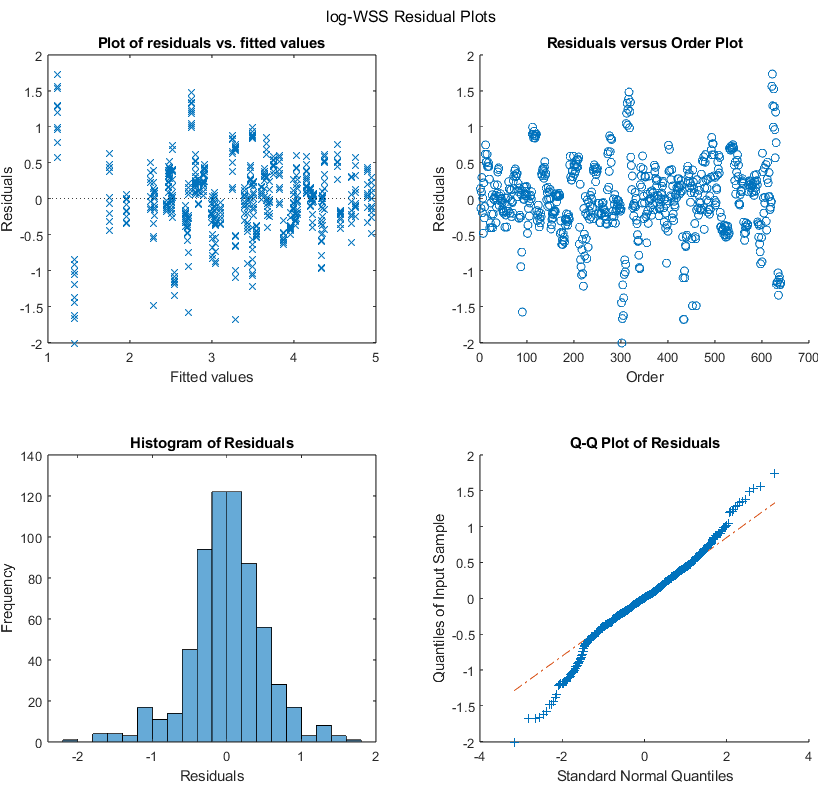


Figure S1.B. Residual plots for the WSS model after log transformation. Residuals appear more symmetrically distributed and homoscedastic compared to the raw model. However, slight curvature in the Q-Q plot and boundary effects remain, suggesting limited improvement in normality and linearity.

**(C) Normalised WSS Model [AIC = -1645.2]**


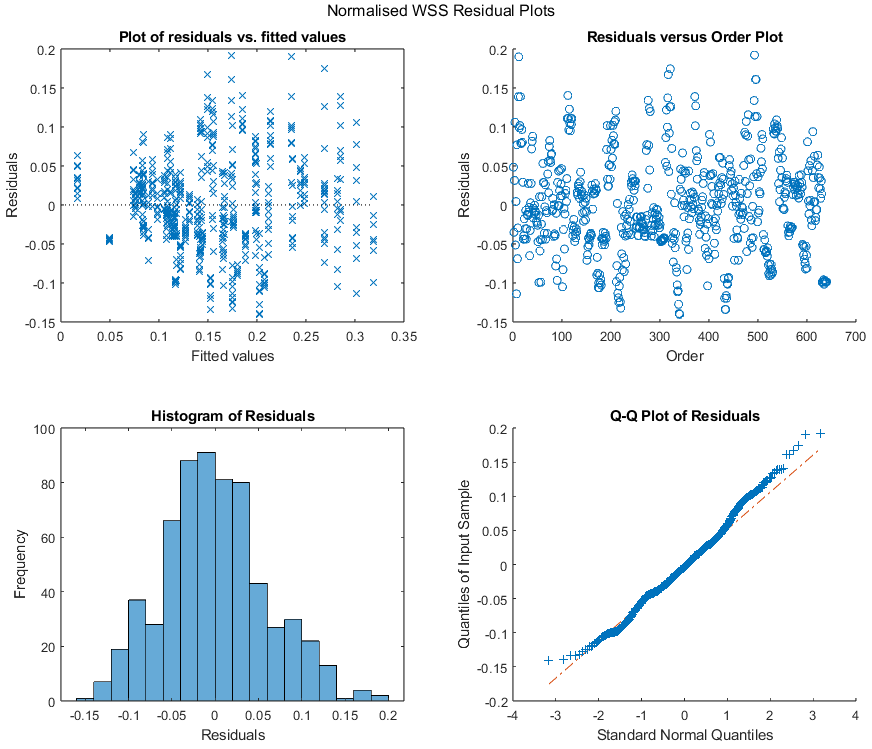


Figure S1.C. Residual plots for the WSS model using normalised values (scaled by patient-specific maximum). The residuals display good symmetry and homoscedasticity, and the Q-Q plot closely aligns with the normal distribution. This transformation yielded the best residual behaviour and was used for final model analysis.
